# Mobile bearing versus fixed bearing medial unicompartmental knee replacement: an independent two center matched-pairs analysis

**DOI:** 10.1007/s00402-022-04629-5

**Published:** 2022-09-28

**Authors:** Mustafa Hariri, Georg Hauer, Maria Smolle, Patrick Sadoghi, Andreas Leithner, Benjamin Panzram, Christian Merle, Tobias Renkawitz, Tilman Walker

**Affiliations:** 1 Clinic for Orthopaedics, University of Heidelberg, Schlierbacher Landstrasse 200a, 69118 Heidelberg, Germany; 2 Department of Orthopaedics and Trauma, Medical University of Graz, Graz, Austria

**Keywords:** Unicompartmental knee replacement, UKR, Knee arthroplasty, Fixed-bearing, Mobile-bearing

## Abstract

**Introduction:**

The aim of the present study was to compare clinical outcome between patients following fixed-bearing (FB) or mobile-bearing (MB) unicompartmental knee replacement (UKR) for antero-medial knee osteoarthrosis (OA) at two independent orthopedic centers.

**Materials and methods:**

Matched-pairs were built between 35 patients receiving FB-UKR and 52 patients following MB-UKR regarding age at time of surgery, body mass index (BMI) and range of motion (ROM) preoperatively. Clinical and functional outcome was measured postoperatively by the American Knee Society Score (AKSS-O/AKSS-F), ROM, Tegner Activity Scale (TAS) as well as the Short Form 36 Health Survey (SF-36).

**Results:**

The average treatment effect of the treated (ATT) after propensity score matching showed a significantly superior ROM in patients following MB-UKR (FB: 118°, MB: 124°). All remaining parameters had no statistically significant differences between both groups involving TAS, AKSS and SF-36.

**Conclusions:**

The present study suggests that MB-UKR can provide a greater ROM compared to FB-UKR on comparable patients. The authors believe that both designs are suitable for adequate improvement of clinical outcome and ROM for patients suffering from antero-medial osteoarthrosis of the knee joint.

**Level of evidence:**

Retrospective cohort study, Level IV.

## Introduction

Antero-medial knee osteoarthrosis (OA) can be treated either with total (TKR) or unicompartmental knee replacement (UKR). In approximately 50% of patients requiring knee arthroplasty, the criteria for using UKR are satisfied [1]. The usage of medial UKR has increased over the last decades with excellent outcomes in patient-reported outcome measures (PROMs) [2–4]. Major advantages of UKR compared to TKR comprises a significant lower rate of adverse events such as infections, blood loss or myocardial infarction [5]. Furthermore, patients following UKR tend to recover faster with shorter hospital stays and better range-of-motion (ROM) compared to patients following TKR [6].

Despite these advantages revision rates of UKR are up to five times higher than TKR, especially in registry-based studies [7, 8]. These differences in survival rates are often attributed to the surgeons learning curve. Liddle et al. showed that the revision rate in medial UKR diminishes with the increase in the number of UKRs performed by a single surgeon per year [5]. Still, these variation of survival rates between independent series, inventor studies and registries highlights the ongoing debate over which arthroplasty is superior.

In principle, there are two different implant design concepts for UKR. The first successful UKR used in 1969 was a fixed-bearing (FB) design with a flat all-polyethylene tibia [9]. To avoid high wear rates due to low conformity between articular surfaces, Goodfellow and O’Connor developed a prosthesis with a mobile-bearing design (MB) [10]. Since the early days of UKR, there has been a continuous improvement in MB- and FB-UKR to reduce design specific disadvantages which subsequently led to good clinical outcomes and survivorship with both implant designs [11, 12].

MB-UKR is designed to replicate tibiofemoral biomechanics better than FB-UKR [13, 14]. Furthermore, the higher conformity of the articular surfaces is considered to reduce contact stress and, therefore, minimize polyethylene wear [15–17]. On the other hand, there are studies which indicate the opposite regarding in vitro wear analyses with less wear rates in FB-UKR compared to MB-UKR [18, 19]. Bearing dislocation is a specific complication in MB-UKR which might lead to early failure of the implant. [20]. Some causes of dislocation are component malposition, release of the medial collateral ligament (MCL) or flexion–extension imbalance [21]. To decrease the risk of dislocation, surgeons tend to perform overcorrection to valgus to offload the medial compartment [22] which may lead to an increasing risk of progressive lateral compartment osteoarthritis [23], whereas an undercorrection of varus deformity is associated with a higher rate of bearing dislocation [24].

Conclusively surgeons need to follow a precise alignment and ligamentous balancing to reduce the risk of failure in MB-UKR, which indicates this design to be more prone to surgeon related errors. Therefore, the revision rate of MB-UKR correlates more with surgical experience and usage of UKR [5, 25, 26]. In FB-UKR the most common modes of failure are polyethylene wear and progressive OA in late years [24, 27].

Despite these differences in modes of failure and survivorship, a variety of studies suggesting similar functional outcomes between both designs [20, 27, 28]. Most of these publications did not consider preoperative parameters as a decisive part on postoperative functional outcome.

In this study we performed a matched-pairs analysis to compare clinical results in patients who underwent MB- or FB-UKR for antero-medial OA at two independent high-volume centers. Our hypothesis was that MB-UKR would be superior with respect to clinical parameters and range of motion (ROM).

## Materials and methods

This matched-pair study is based on the retrospective analysis of prospectively collected data of a series of patients which underwent UKR for antero-medial OA in two different orthopedic centers. In total 272 UKR were analyzed subdivided into 35 FB-UKR implanted in the Department for Orthopaedics and Trauma of the University of Graz, Austria (Group A) and 237 MB-UKR implanted in the Center for Orthopaedics and Trauma Surgery of the University of Heidelberg, Germany (Group B). In group A, the Sigma® High Performance (HP) Partial Knee System (DePuy Synthes, Warsaw, IN) was used as FB-UKR and the Oxford Phase III Prosthesis (Biomet UK Limited, Swindon, UK) was used as MB-UKR in group B. In both centers, the primary indication was advanced osteoarthritis of the medial compartment with full-thickness articular cartilage loss or avascular necrosis of the medial femoral condyle. In all cases, the anterior cruciate ligament (ACL) as well as the medial (MCL) and lateral (LCL) collateral ligaments were functionally intact, the varus deformity was manually correctable at 20° flexion and there was no evidence of osteoarthritis in the lateral compartment on valgus stress radiographs. Osteoarthritis of the patellofemoral joint was not considered to be a contraindication unless there was deep eburnation or bone grooving on the medial facet of the patella.

In both centers, all surgeries were performed using the minimally invasive surgical technique through a medial parapatellar approach without dislocation of the patella. Whereas, all FB-UKR were implanted with cemented fixation, MB-UKR were implanted with cemented or uncemented fixation depended on the bone quality. An intravenous single-shot antibiotic (1.5 g cefuroxime) was administered perioperatively. Immediate full weight bearing was allowed postoperatively.

At the last follow-up, the clinical outcome was evaluated using the American Knee Society Scores (AKSS-F and AKSS-O) [29] as well as the range of motion (ROM). ROM was objectified by goniometer pre- and postoperatively. Postoperative activity level was assessed using the Tegner Activity Scale (TAS) [30]. The state of general health was assessed using the Short Form 36 Health Survey (SF-36), which is a widely used assessment due its reliability and validity [31, 32].

### Statistical methods

All data were analysed by Stata Version 17 (Stata Corp LLC, Texas, USA).

Means and medians are provided with corresponding standard deviations and interquartile ranges for normally and non-normally distributed variables, respectively. Propensity score matching using the Stata add-on psmatch2 was used to account for imbalances between the two cohorts. A propensity score (PS) was calculated based on patient age at time of surgery, body mass index (BMI), and ROM of the affected joint at time of surgery. Nearest neighbour-matching with 3 neighbours was used to calculate the matched outcome was applied. Balancing of variables between matched cohorts was assessed with Stata add-on pstest. Remaining bias of < 5% was considered as sufficient balancing. The average treatment effect of the treated (ATT) was assessed for specific outcome variables on the matched and PS-weighted cohort. A *p* value of < 0.05 was considered statistically significant.

An a priori power analysis according to the magnitude of a 10% difference for the endpoints AKSS-F, AKSS-O and ROM was performed with a *p* value < 0.05 and a power greater than 80%, which revealed a minimum number of *n* = 35 cases per group as sufficient. Matching was performed with respect to age, BMI, and preoperative ROM in a 1:1.5 ratio and the maximum caliper set at 0.2.

## Results

We were able to match 35 patients following FB-UKR (Group A) with 52 patients following MB-UKR (Group B) regarding parameters age at time of surgery, BMI and ROM preoperatively. Hereby, we eradicated significant differences between both groups preoperatively in those mentioned parameters after Propensity-Score matching (*p* > 0.05, Table 1).

**Table 1:** Propensity score matching for both groups

Parameter	Sample	FB-UKR	MB-UKR	*p* value
Age	Unmatched	66.03	56.76	< 0.001
	Matched	66.03	66.27	0.912
BMI	Unmatched	28.61	30.72	0.034
	Matched	28.61	28.66	0.936
ROM	Unmatched	105.43	121.46	< 0.001
	Matched	105.43	108.90	0.389

*BMI* Body mass index, *ROM* range of motion, *FB* fixed-bearing, *MB* mobile-bearing, *UKR* unicompartmental knee replacement

The matched pairs are well balanced in age (bias − 2.5%) and BMI (bias − 1.7%) such as moderate balanced in ROM preoperatively (bias − 22.0%). Patient demographic data are shown in Table 2.

**Table 2:** Demographics

Demographics	
Patients	87 (35 FB-UKR, 52 MB-UKR)
Sex (m/f) (*n*/%)	33 (37.9%)/54 (62.1%)
Mean age at surgery in years (SD)	64.8 (9.6)
Mean age at last follow-up in years (SD)	68.0 (8.9)

*SD* standard deviation, *FB* fixed-bearing, *MB* mobile-bearing, *UKR* unicompartmental knee replacement

The ATT after propensity score matching showed a significantly superior postoperative ROM in Group B (FB: 118.43, MB: 124.86, t-score: − 2.55). All remaining parameters had no statistically significant differences between groups involving TAS (FB: 3.97, MB: 3.62, t-score: 1.46), SF-36 items function (FB: 85.71, BB: 77.38, t-score: 1.77), role function (FB: 78.75, MB 75.24, t-score: 0.44), pain (FB: 78.00, MB: 75.24, t-score: 0.24), condition (FB: 71.54, MB: 67.10, t-score: 0.88), vitality (FB: 68.21, MB: 66.90, t-score: 0.29), social function (FB: 91.07, MB: 88.45, t-score: 0.60), emotional (FB: 83.57, MB: 84.44, t-score: − 0.12), psychologic (FB: 77.29, MB: 81.71, t-score: − 1.12), such as AKSS-F (FB: 93.14, MB: 89.19, t-score: 1.19). The AKSS-O was slightly better in group A (FB: 96.86, MB: 91.84, t-score: 1.90). All details are given in Table 3.

**Table 3:** Clinical results before and after propensity score matching

Parameter	Sample	FB-UKR	MB-UKR	Difference	t-score
ROM	Unmatched	118.43	126.14	− 7.71	− 4.48
	Matched	118.43	124.86	− 6.43	− 2.55*
TAS	Unmatched	3.97	3.70	0.27	1.34
	Matched	3.97	3.62	0.35	1.46
SF-36 Function	Unmatched	85.71	74.47	11.24	2.77
	Matched	85.71	77.38	8.33	1.77
SF-36 Role function	Unmatched	78.75	73.42	5.33	0.79
	Matched	78.75	75.24	3.51	0.44
SF-36 Pain	Unmatched	78.00	70.71	7.29	1.42
	Matched	78.00	76.30	1.70	0.24
SF-36 Condition	Unmatched	71.54	64.15	7.39	2.03
	Matched	71.54	67.10	4.44	0.88
SF-36 Vitality	Unmatched	68.21	63.73	4.48	1.30
	Matched	68.21	66.90	1.31	0.29
SF-36 Social function	Unmatched	91.07	85.76	5.31	1.36
	Matched	91.07	88.45	2.62	0.60
SF-36 Emotional	Unmatched	83.57	82.56	1.01	0.17
	Matched	83.57	84.44	− 0.87	− 0.12
SF-36 Psychologic	Unmatched	77.29	76.96	0.32	0.10
	Matched	77.29	81.71	− 4.42	− 1.12
AKSS-F	Unmatched	93.14	89.30	3.84	1.37
	Matched	93.14	89.19	3.95	1.19
AKSS-O	Unmatched	96.86	89.44	7.42	3.38
	Matched	96.86	91.83	5.03	1.90

*ROM* range of motion, *TAS* Tegner Activiy Scale, *SF-36* Short form 36 health survey, *AKSS* American Knee Society Score, *FB* fixed-bearing, *MB* mobile-bearing, *UKR* unicompartmental knee replacement

*p < 0.05

## Discussion

The aim of our study was to compare clinical outcome parameters of patients following MB-UKR and FB-UKR for antero-medial OA in a matched pairs two center study. Our hypothesis was that MB-UKR would be superior with respect to clinical parameters and ROM.

The most important finding of the study is that ROM after MB-UKR seems to be significantly superior than after FB-UKR. As our patients were matched regarding age, BMI and preoperative ROM, it is assumable that there was no influence on differences regarding the clinical outcome between both groups by these parameters.

While many studies analyzed survivorship of FB- and MB-UKR and only reported second about functional outcome parameters, this present study focused primary on the clinical outcome of those designs [17, 20, 27, 28]. Interestingly to date there are only 3 randomized control trials (RCT) comparing outcome parameters of both implant designs [33–35]. While two are outdated [34, 35], the remaining study compared a robotic-assisted procedure in FB-UKR with a conventional implantation of MB-UKR [33], which might influence the postoperative functional outcome parameters regardless the used implant design. Despite these studies, the mentioned publication were mostly systematic analyses comparing functional outcome parameters between both implant designs without taking the preoperative status into account. In contrast to this, our patients were matched by the preoperative knee function, which is a strength of the current study.

Gleeson et al. reported about no differences in ROM after MB-UKR and FB-UKR as did Li et al. comparing the Miller-Galante Unicompartmental Knee (FB-UKR) with the Oxford UKA (MB-UKR) [35, 36]. Furthermore, Parratte et al. compared 79 FB-UKR with 77 MB-UKR demonstrating no statistically significant differences regarding postoperative ROM in both groups [37]. To our knowledge, the present study is the first matched-pair analysis between FB-UKR and MB-UKR using preoperative ROM as a matching parameter to exclude any influence on the postoperative outcome by this parameter. Therefore, we suggest MB-UKR can provide a greater ROM than FB-UKR considering the preoperative ROM. This might be explained by a more physiologically joint kinematic after MB-UKR as reported by Li et al. [14, 38]. Nevertheless, the clinical benefit of the superior ROM remains unsure as it is not reflected in the functional outcome and activity level in our cohorts.

There was no statistically significant difference in the postoperative AKSS between both groups, while both achieving excellent results comparable to other studies [20, 39–41].

The current literature supports the hypothesis that clinical results following FB-UKR and MB-UKR are comparable and do not differ significantly. A systematic review by Peersman et al. analyzing 44 studies reported similar AKSS-O/ AKSS-F Scores after 5 years for FB-(88.2/86.4) and MB-UKR (88.1/85.7)[20]. A randomized prospective study comparing both designs demonstrated good to excellent clinical results regarding the AKSS without differences between them [36]. Supporting these findings Smith et al. found no differences between FB- and MB-UKR groups regarding the Bristol Knee Score, the Western Ontario and McMaster Universities Osteoarthritis Index (WOMAC) score, Oxford Knee Score (OKS) and AKSS in their systematic analysis [28].

Since the usage of UKR increased in the last decade, consequently more young and active patients are treated with UKR [42, 43]. As clinical results are not always conclusive with the actual activity level of the patient [44], it is helpful to use activity rating scales such as the TAS additionally. Earlier studies have shown that patients following FB- and MB-UKR can restore their activity level, especially in low impact sports such as hiking, swimming and cycling [11, 45–47]. While several studies reported differences in postoperative activity levels between patients following TKR and UKR, there are no studies that have shown such differences between patients following FB- and MB-UKR [8, 45, 48, 49]. In our study, there was no difference in postoperative activity level measured by TAS between Group A (3.97) and Group B (3.62), which supports the hypothesis that both implant designs can provide a moderate level of activity postoperatively.

Regarding the general state of health of patients following UKR, Pronk et al. have shown similar results for both implant designs assessed by the 3-level version of EuroQol 5 Dimensions [50]. Li et al. reported a significant improvement of quality of life measured by the SF-36 Score from preoperative to 2 years postoperatively in 28 FB- and 28 MB-UKR without any difference between the groups [36]. Consistent with that, Neufeld et al. have shown no statistically significant difference after a 10 year follow-up regarding the SF12-Score between patients following FB- and MB-UKR [40]. Our matched-pairs analysis fits in the current literature as it showed no significant differences in the general state of health between both groups measured by the SF-36 Score.

There are several limitations to this study such as the limited sample size especially in the FB-group as well as the retrospective study design. In addition, functional outcome parameters were only assessed postoperative. Therefore, a comparison between changes from preoperative to postoperative were not possible.

Since the ROM was assessed with a goniometer by many different examiners, there could be a higher interobserver variability in our study design. Accordingly, the shown differences in ROM between both groups need to be carefully interpreted. Even though the ROM may differ statistically significant, the authors believe it is not relevant in this extent regarding patient benefits.

In addition, two different systems implanted by different surgeons were evaluated in this analysis. However, both systems are only available in the used designs, and therefore, a comparison between different developers is inevitable to analyze design specific differences. Due to a matched-pair and propensity score analysis, we could further minimize this bias.

## Conclusions

This matched-pairs analysis showed a significantly superior ROM after MB-UKR compared with FB-UKR, while there were no significant differences in AKSS, TAS and SF-36. The authors believe that both designs are suitable for adequate improvement of clinical outcome and ROM for patients suffering from antero-medial osteoarthritis of the knee joint.

## Data Availability

The data sets used and analyzed during the current study are available from the corresponding author on reasonable request.

## Abbreviations

ACL: Anterior cruciate ligament
AKSS: American knee society score
ATT: Average treatment effect of the treated
BMI: Body mass index
FB: Fixed-bearing
LCL: Lateral collateral ligament
MB: Mobile-bearing
MCL: Medial collateral ligament
OA: Osteoarthrosis
OKS: Oxford knee score
PROMs: Patient-reported outcome measures
RCT: Randomized control trials
ROM: Range of motion
SF-36: Short Form 36 Health Survey
TAS: Tegner Activity Scale
TKR: Total knee replacement
UKR: Unicompartmental knee replacement
WOMAC: Western Ontario and McMaster Universities Osteoarthritis Index
